# mHealth Series: Measuring maternal newborn and child health coverage by text messaging – a county–level model for China

**DOI:** 10.7189/jogh.03.020402

**Published:** 2013-12

**Authors:** Yanfeng Zhang, Li Chen, Michelle H. M. M. T. van Velthoven, Wei Wang, Li Liu, Xiaozhen Du, Qiong Wu, Ye Li, Josip Car

**Affiliations:** 1 Department of Integrated Early Childhood Development, Capital Institute of Pediatrics, Beijing, China; 2 Global eHealth Unit, Department of Primary Care and Public Health, Imperial College London, London, United Kingdom; 3 Department of International Health, Johns Hopkins Bloomberg School of Public Health, Baltimore, Maryland, USA

## Abstract

**Background:**

Effective interventions in maternal, newborn and child health (MNCH), if achieving high level of population coverage, could prevent most of deaths in children under five years of age. High–quality measurements of MNCH coverage are essential for tracking progress and making evidence–based decisions.

**Methods:**

MNCH coverage data are mainly collected through fieldworkers’ interview with preselected households in standard programs of Demographic and Health Surveys (DHS) or Multiple Indicator Cluster Surveys (MICS) in most low– and middle–income countries. Household surveys will continue to be the major data source for MNCH coverage in the foreseeable future. However, face–to–face data collection broadly used in household surveys is labor–intensive, time–consuming and expensive. Mobile phones are drawing more and more interest in medical research with the rapid increase in usage and text messaging could be an innovative way of data collection, that is, we could collect DHS data through mHealth method. We refer to it as “mDHS”.

**Finding:**

We propose in this paper a conceptual model for measuring MNCH coverage by text messaging in China. In developing this model, we considered resource constraints, sample representativeness, sample size and survey bias. The components of the model are text messaging platform, routine health information system, health facilities, communities and households.

**Conclusions:**

Measuring MNCH interventions coverage by text messaging could be advantageous in many ways and establish a much larger evidence–base for MNCH health policies in China. Before mDHS could indeed be launched, research priorities would include a systematic assessment of routine health information systems and exploring feasibility to collect name lists, mobile phone numbers and general demographic and socio–economic data; qualitative interviews with health workers and caregivers; assessment of data validity of all indicators to be collected by text messaging; and exploring approaches to increase participation rate.

Globally, in 2011 seven million children died before reaching their fifth birthday [1]. Effective interventions, if reaching all children in need, could prevent most of these deaths [2]. To maximize the effectiveness, interventions should be delivered under the principle of continuum of care for mother and child, that is, from pregnancy, through birth, the newborn period, infancy and childhood. They should achieve the highest possible level of coverage [3]. Intervention coverage in maternal, newborn and child health (MNCH) is defined as the proportion of children under 5 years of age (or their caregivers, or pregnant women) in the population who needed the intervention and have actually received it [3]. Effective measurements of MNCH intervention coverage are very important to decision makers, program managers and donors for tracking the progress and making evidence–based decisions.

In most low– and middle– income countries, health management information systems are currently too weak to provide data of adequate quality for assessing and guiding health programs [4,5]. Therefore, data on MNCH coverage in these countries are mainly generated through household surveys in international standard programs of Demographic and Health Surveys (DHS) [6] or Multiple Indicator Cluster Surveys (MICS) [7]. Neither DHS nor MICS program is being implemented in China, and national surveys, such as National Health Services Survey and National Nutritional and Health Survey, are the major sources of MNCH coverage data [8,9]. An effective data collection method is essential for household surveys. Face–to–face interview with preselected households by trained field workers is broadly used around the world. This method, however, is labor–intensive, time consuming and expensive [10]. Nowadays, mobile technologies are increasingly drawing interest in medical research with the rapid increase of mobile phone use and text messaging could be an innovative way of data collection [11]. Text messaging does not require field visits. It is accessible for most people and offers an opportunity for real time data collection and no interviewer bias [10].

The recently published “Measuring Coverage in MNCH” Collection by *PLOS Medicine* [12] systematically assessed the validity of intervention coverage measurement based on household surveys. The studies showed that measurements of some indicators used to track intervention coverage may not be fully reliable [12]. In their strategies for improving coverage measurement through household surveys, “Incorporate information technology” was proposed and eHealth and mHealth were deemed as having important implications for coverage measurement and monitoring [12]. Technology can improve data quality in household surveys and is increasingly being used in real–time measurement of child health program indicators [13].

This article proposes a concept model of measuring MNCH coverage by text messaging at the county level in China. Using Zhao County, Hebei Province as a case study, we discuss why to measure MNCH coverage by text messaging, along with the challenges and future research agenda.

## WHY MEASURE MNCH COVERAGE BY MOBILE TEXT MESSAGING

The *PLOS Medicine* Collection concluded that household surveys will continue to be the major data source for MNCH coverage in the foreseeable future [12]. However, DHS, MICS and other household surveys have significant problems that need to be addressed, such as, how to ensure that household surveys produce the best and most relevant information [12]. Some of these problems are inherently caused by the current data collection method of household surveys, that is, visiting households by trained fieldworkers to collect data using pen–and–paper or handheld electronic devices.

### Resource constrains

Regular household surveys carried out according to minimum standards are required to provide frequent and high–quality coverage data for program monitoring [14]. However, in current household surveys (visiting households by fieldworkers to conduct face–to–face interview), recruitment, training, transportation and accommodation of interviewers and supervisors are very labor–intensive, time consuming and expensive, and hence a major constraint to frequent conduction of household surveys, especially in resource–limited settings. Using text messaging can achieve remote data collection and dramatically reduce personnel, time and cost. This could be invested either in increasing the coverage or used as an incentive for participants.

### Sample representativeness

Most household surveys are sample surveys in which representative samples are preselected with each household having a known chance to be selected. Response rates are crucial because low response rates can damage the representativeness of the sample. However, high response rates are not easily achieved in the field since caregivers are often unavailable due to work outside home in day time or being too busy to be interviewed. Thanks to multiple revisits to households and close monitoring of response rates by field staff, non–response in DHS and MICS is well below 10% in most countries [15], but resource constrains need to be considered. In addition, poor transportation and security concerns prevent interviewers from visiting households in some circumstances. Experience indicated that interviewers have a tendency to modify children’s age, such as transferring children to age group of over 5 years, to exclude them from the survey sample and thereby reduce interviewers’ workload [15]. Moreover, some cultural or custom factors prevent fieldworkers to visit households. For example, in rural China, people believe that it is not good for newborns and mothers to be visited by other people within the first months of birth [16]. Therefore, fieldworkers cannot go to households which often results in fewer newborns in an actual survey sample. Text messaging survey could overcome these problems as no household visits would be needed, potentially leading to increased representativeness of hard–to–reach populations, as all participants can respond at the time of their convenience.

### Sample size

Sample size is another issue to be considered. An increased sample size is needed for disaggregated analysis by sex, age and socioeconomic status, and for indicators suffering from seasonality issues, such as prevalence of suspected pneumonia and diarrhea, because results from one survey in a year only represent a specific season and cannot provide a full picture of all seasons [17]. Very large samples are required for obtaining adequate denominators to support coverage measurement for low prevalence events [12]. Currently, DHS and MICS surveys include around 15 000 and 10 000 households respectively and are usually conducted every 5 years [15]. Due to limited resources, it is usually not feasible to conduct household surveys with very large samples or more frequently. Text messages could be sent to as many caregivers as possible to dramatically increase the sample size, and text messaging survey could be frequently conducted, eg, every 3 months or even more frequently, to catch the indicators in various seasons.

### Survey bias

The current household surveys have some biases, such as interviewer bias and recall bias. Both DHS and MICS have minimum requirements for selecting interviewers, eg, having at least a high school diploma and not directly being involved in the management or provision of health services, to avoid potential conflict of interests [15]. In addition, all interviewers are trained on survey protocol and evaluated before conducting field work. However, interviewer bias cannot be completely avoided. In addition, most MNCH coverage indicators are gathered by recall of survey participants, with recall periods exceeding 2 years for some indicators. For example, a mother of 2–year–old child has to recall what happened two years ago when asked about initiation of breastfeeding of her child. A text messaging survey has no interviewer bias and could be conducted in real–time, thus significantly reducing the recall bias.

Mobile phone ownership increased rapidly worldwide, and China is no exception. In May 2013, there were almost as many mobile phone subscriptions as people in the world (estimated 6.8 billion mobile subscriptions) and 1.2 billion subscriptions in China, which equals to 85.9% of the population [18]. Zhao County has a total population of 580 000 and approximately 75% of the population has mobile phones. Nearly all households have at least one mobile phone [19]. Our literature review revealed that an increasing number of studies have explored the feasibility of using text messaging as a data collection method, and found that it was generally accepted by participants, had reasonable agreements and lower costs compared with other methods, and variable response rate [10,11,20–25]. Therefore, text messaging may possess a significant potential for overcoming constrains of face–to–face field interviews, and could be used as an alternative way of measuring MNCH coverage.

## COMPONENTS OF THE MODEL

An appropriate text messaging platform is a prerequisite for measuring MNCH coverage by text messaging. Through sending and receiving messages, the platform builds relations with households. However, as an innovative data collection method, text messaging cannot operate on its own. Resources from routine health information system (HIS), health facilities and communities should be mobilized and utilized to maximize its effectiveness. Therefore, in our model, text messaging platform, routine HIS, health facilities, communities and households have their own roles and should be closely related to each other.

### Information to be collected

In DHS, MICS and most other household surveys, in addition to coverage data, basic information on children and mothers, household information on demographic and social economic status are collected for further analysis, such as inequity analysis [4,6,14]. Maternal, Newborn and Child Health Household Survey (MNCH HHS) developed by the World Health Organization (WHO) also collects data on delivery channels of key interventions and reasons for coverage failures (unpublished data, 2009). All these need to be carefully considered for the feasibility of their collection by text messaging.

### Roles of different partners

**Figure 1:**
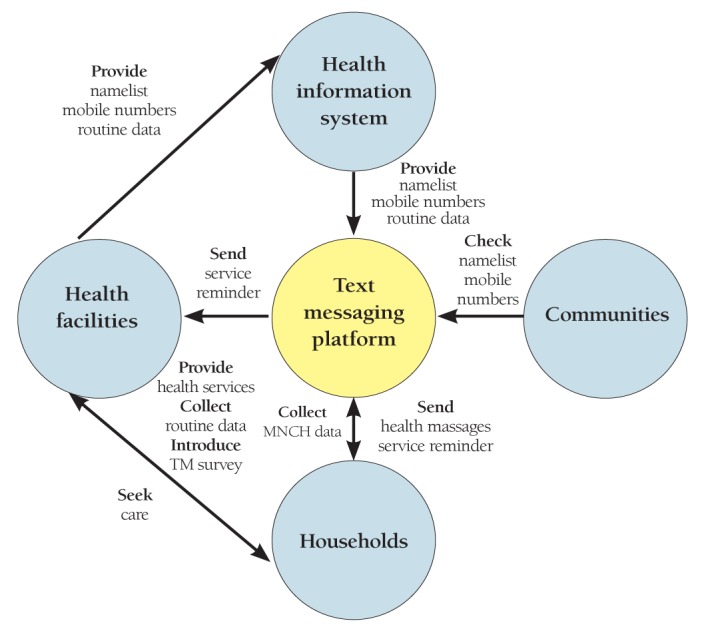
Roles and relationship of different partners in the model.

Roles of different partners are presented in **Figure 1**.

**Text messaging platform.** A text messaging platform consisting of software and management staff is the core of our model. The primary role of the text messaging platform would be to collect data from households by sending survey questions and receiving answers from which the MNCH coverage indicators are calculated. We could also send service reminders and health education messages to households. By explaining when, where, what and why of health services, service reminder messages have the potential to improve the recipients’ recall of interventions they received. Health messages, such as breastfeeding and complementary feeding recommendations, may increase the survey response rate from households because caregivers could benefit from text messaging communications. In addition, text messages could also be sent to health workers to remind them of explaining services to health recipients. Therefore, service reminders and health messages hold a potential to improve the quality of MNCH coverage measurements.

**Routine health information system.** There are many sources of information from routine HIS, such as records of basic public health services for women and children, annual report system, monthly report of births from delivery institutions to the county Maternal and Child Health Hospital, etc. Some of these routine health records have been electronic as required by the basic public health programs, such as health archives, antenatal visits, postnatal visits, and child health care visits. Routine HIS is required to collect such information, therefore, could serve as a source of name lists and mobile numbers, both of which are the basis for text messaging data collection. All these electronic health records could be used to complement and cross–check each other.

**Health facilities.** County–level hospitals, township hospitals and village clinics are the main health facilities in Zhao County. They provide clinical and public health services for the population in their catchment areas. Health facilities can play an important role in measuring MNCH coverage.

First, health facilities are the main sources of routine HIS. The name lists, mobile phone numbers, basic information of children and their caregivers, and demographic and socio–economic information are mainly collected by health workers when providing health services in their facilities. The completeness, accuracy and timeliness of information from routine HIS highly depend on the performance of health workers and facilities. In addition, health facilities can help with the update of name lists and mobile phone numbers at every service contact with children and caregivers.

Second, good communication with service recipients may increase their willingness to respond and improve their recall of interventions. Response rate is a large challenge for a text messaging survey. Introduction of the survey to participants by health workers when providing health services may be an effective way to increase participants’ responses. Recall bias of respondents is an important factor affecting the quality of coverage measurement by household surveys. Service recipients are more likely to forget interventions which they are not familiar with, or do not pay attention to. Therefore, careful explanations by health workers about contents and reasons of interventions being delivered are likely to improve recall [12].

Third, the extent and quality of health services provided by health facilities can help us better estimate coverage of some interventions [12]. For example, blood tests of syphilis, HIV and hepatitis B are the standard services for antenatal care in Zhao County MCH hospital. Pregnant women or mothers often did not know whether or not they had these tests, but they can remember whether or not they went to the county MCH hospital and gave blood samples. Therefore, we can link recipients’ recall and hospital’s standard services to estimate the coverage. Given the potential role to improve coverage measurement held by health facilities, government’s efforts to strengthen health facilities should include quality of care as well as quality of health information.

**Communities.** Community workers, such as family planning workers, are familiar with households and births, and therefore could be used to check and validate the name lists and mobile phone numbers. They could also help with explanation of text messaging to caregivers.

**Households.** Households are the information source and crucial to the quality of data collected. They have direct contact with health facilities through care–seeking and with the text messaging platform through receiving and replying to text messages. Efforts are needed to familiarize households with text messaging survey.

### Operation guidelines

**Figure 2:**
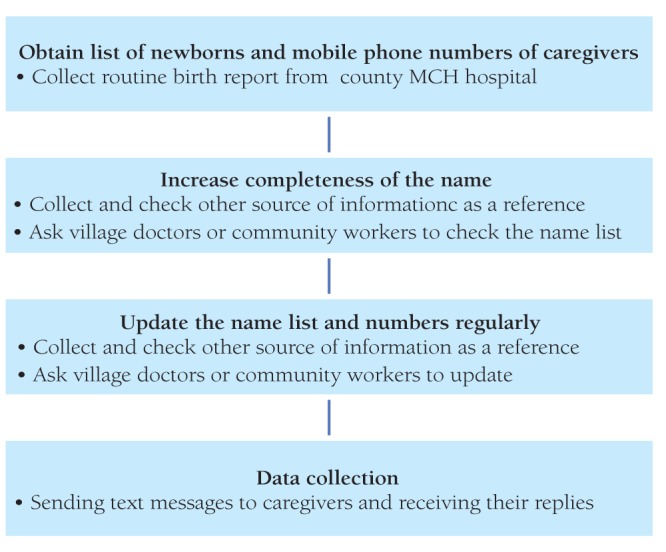
Procedure of text messaging data collection.

Guidelines for operation of the programme are presented in **Figure 2**. Sample representativeness is essential for the accurate measurement of the MNCH coverage. To obtain a representative sample, a complete and accurate name list of target children needs to be acquired. Then, those name lists need to be checked and updated regularly, because children living in an area may migrate in and out or die. Mobile phone numbers of caregivers are prerequisite to text messaging data collection, and should be obtained at the same time as name lists. In addition, mobile phone numbers need to be validated and updated regularly. With the name lists and mobile phone numbers ready, data collection could begin.

Children can be delivered in township hospitals, county level hospital, hospitals outside the county or outside health facilities (eg, at home). All delivery facilities in Zhao County are required to report all deliveries to the county MCH hospital every month (Deliveries outside the county or outside health facilities may be missed). In Zhao County, 99.5% of newborns were delivered in hospitals (unpublished data), therefore, delivery institutions could be a good point for engagement of caregivers. They could be provided with information materials, explaining the purpose of the mHealth communications platform which will be used for data collection, and be educated on how to answer survey questions. Then we could collect routine information on birth from county MCH hospital every month to obtain name lists of newborns.

Since some births will be missed, other sources of information may be used to increase the completeness of the namelists. Possible routine HIS sources include antenatal visits of pregnant women from county level hospitals and township hospitals, annual report systems, and immunization records. In addition, routine HIS, village doctors and community workers can be used to update the namelists regularly.

Mobile phone numbers can be collected and updated by the same mechanism as name lists. However, mobile phone numbers collected are sometimes wrong due to mistakes of caregivers, health workers or data input, and therefore they have to be validated. To achieve this, text messages can be sent to all numbers and mobile phone holders can be asked to reply. Health workers and community workers can help with correction of wrong numbers

## CHALLENGES

Although text messaging has a significant potential to be used to measure MNCH coverage in household surveys, there are many challenges which need to be addressed, including completeness and accuracy of children’s name lists and caregivers’ mobile phone numbers, data validity and response rate.

### Completeness and accuracy of children’s name lists and caregivers’ mobile phone numbers

Households are the sample unit in DHS and MICS programs. In our model, children would be the sample unit and therefore, name lists of children have to be obtained through routine health information systems. The quality of routine health information systems is often low in low– and middle–income countries as well as in China [4,5], that is, the name lists are often incomplete and inaccurate. Mobile phone numbers would be the prerequisite to conduct text messaging surveys and would also be collected through routine health information systems. Data from different sources could be complemented and cross–checked with each other. However, the extent of the completeness and accuracy are still unknown.

### Data validity

Data validity is an essential issue for any new method of collection to be accepted. Therefore, data collected by text messaging has to be validated before this new method is used to measure MNCH coverage. As indicated in recent studies, sensitivity and specificity of current coverage indicators measured by face–to–face interview are highly variable across interventions, with some indicators being very low [12]. This problem would also occur in a text messaging survey, or even worsen because caregivers may not understand the survey questions adequately due to limitations of length of messages, or lack of communication between interviewers and caregivers as in face–to–face survey. Moreover, answers to the survey questions are recorded by trained interviewers in face–to–face interviews, whereas in text messaging surveys, they would be entered by caregivers themselves, which may not be as standard as in face–to–face interviews.

### Low response rate

Response rate is crucial to sample representativeness and sample size of household surveys. Non–response rate of household surveys in DHS and MICS could be controlled below 10%. However, it would be very difficult for a text messaging survey to achieve such a high response rate. As indicated in literature, response rate of text messaging data collection varied from 6% to 100% [10,11,20–25]. Our studies in Zhao County showed low response rate of around 30% (our unpublished data). Increasing response rate of text messaging surveys is a big challenge in our model and multiple approaches need to be explored and tested in further studies.

## FUTURE RESEARCH

Facing all challenges above, further research is needed to explore solutions to possible problems. Proposed research priorities are summarized below.

### **Systematic assessment of routine HIS and exploring feasibility to collect name lists, mobile phone numbers and general demographic and socio**–**economic data**

The routine HIS in Zhao County include: 1) antenatal care visit to county MCH hospital; 2) antenatal care visit to township hospitals; 3) annual report system; 4) monthly birth report from delivery institutions to county MCH hospital; 5) contact with township hospitals for getting hospital delivery subsidies; 6) child health care visits to township hospitals; 7) namelists for immunization. We will systematically assess contents, mechanism and quality of these routine HIS and discuss the feasibility to collect namelists, mobile phone numbers, demographic and socio–economic data (see Box 1 for information to be assessed).

Box 1 **Data to be assessed in routine Health Information Systems**

Who is responsible?

How are data collected?

Advantages and disadvantages of different data sources

Quality of data from different sources

What information is included?

Possibility to add or reduce information

Relations of different data sources

Possibility to integrate different data sources

Electronically or not?

Mobile phone numbers included?

Current mechanisms for dynamic update?

Any others?

### Qualitative interviews with health workers and caregivers

Health workers and caregivers would be essential to text messaging surveys; therefore, it is important to understand their willingness to be involved and their attitude and possible preference towards using text messaging. Qualitative studies are therefore needed to explore their mobile phone use behaviour, and to obtain their opinions on text messaging surveys and possible problems and solutions.

### Validating name lists and mobile phone numbers and exploring mechanisms of regular update of information

A household census should be conducted in some selected townships and villages to validate the name lists and mobile phone numbers collected by routine HIS. Using household census as gold standard, by comparison, could reveal to what extent data obtained from routine HIS are complete and accurate. Caregivers could also be interviewed during household census to explore the mechanism of regular update of the information.

### Data validity

Coverage indicators to be measured in our model would cover antenatal care, delivery and postnatal care, infant and young child feeding practices and prevalence and care–seeking of pneumonia and diarrhea. Studies are needed to assess data validity of all indicators to be collected by text messaging. A standardized study protocol needs to be developed and tested, and the methodologies described in papers of *PLOS Medicine* Collection should be referred to.

### Increase response rate

There are many factors which may affect the response rate of text messaging data collection, such as an advance letter, acquiring trust of participants, increasing attempts, targeting sending time, money incentives, providing health education messages, etc. [11,25–27]. Further studies need to be conducted to test the effectiveness of these approaches on increasing response rate of text messaging surveys.

### Real measurement of MNCH coverage

A pilot real measurement of MNCH coverage is needed to test how to operate and problems encountered should be systematically recorded. Possible solutions to problems would also be explored and tested to further improve the model.

In this paper, we proposed a county–level conceptual model of using text messaging as a data collection tool to measure MNCH coverage in China. Text messaging may hold a great potential to be used in household survey. However, major challenges need to be addressed and further researches need to explore the feasibility of the model.
